# Empirical Analysis of Retirement Pension and IFRS Adoption Effects on Accounting Information: Glance at IT Industry

**DOI:** 10.1155/2014/809219

**Published:** 2014-06-09

**Authors:** JeongYeon Kim

**Affiliations:** Sangmyung University, 20 Hongjimun 2-gil, Jongno-gu, Seoul 110-743, Republic of Korea

## Abstract

This study reviews new pension accounting with K-IFRS and provides empirical changes in liability for retirement allowances with adoption of K-IFRS. It will help to understand the effect of pension accounting on individual firm's financial report and the importance of public announcement of actuarial assumptions. Firms that adopted K-IFRS had various changes in retirement liability compared to the previous financial report not based on K-IFRS. Their actuarial assumptions for pension accounting should be announced, but only few of them were published. Data analysis shows that the small differences of the actuarial assumption may result in a big change of retirement related liability. Firms within IT industry also have similar behaviors, which means that additional financial regulations for pension accounting are recommended.

## 1. Introduction

Many developed countries have introduced several types of pension scheme for wage earners to diminish expected economic difficulties after their retirement. Occupational pension scheme, which is introduced recently in Korea, organizes 3-tier social security program with national pension and individual pension service. It can be differentiated from others by the fact that the responsibility of retirement pension payment is on related company.

Historically, the issue of retirement allowance from the workers' view point is the reliability of payment [1]. Government tries to guarantee retired employees the payment of predefined retirement allowances, but there are many overriding legal considerations for the bankrupted companies' obligations. Besides, new pension scheme as a retirement allowance raises other questions from the firm's accounting view point on how to describe pension related liabilities in firm's financial reports. Pension is a kind of future payment and its exact amount is not predictable.

To improve above issues, Korean government introduced important changes in retirement allowance policy and related accounting standards. First, Korean government required that all listed companies entrust predefined retirement allowances to selected financial organizations. The responsibility of retirement allowance is still on each company, but new policy ensures the minimum payment of it with the reserved money. Second, Korean government required that all listed companies provide workers with several pension schemes as a retirement allowance and report related liability based on K-IFRS in their financial reports.

There are many researches on the effectiveness of new retirement allowance policy checking if the policy actually helps retired workers to get their retirement allowance timely or if the external funds are properly operated by the financial organizations for payments. In addition to them, there are arguments about the effects of new pension scheme on individual financial reports. Logically, it may give no effects on the business results of firms. New policy just requires that companies transit internal reserves for retirement allowance into an external organization.

However, new financial standards with pension accounting make remarkable changes in liability estimation method for retirement allowances. New financial standards, K-IFRS, take a principle-based approach without guidelines for operational details and allow firm's alternation within given principles. Pension accounting needs complicated projection process for the expected liability with several actuarial assumptions. The guideline for the required variables for the assumptions such as life expectancy or average wage increase is provided, but there are no specific regulations yet in Korea.

Researchers reported that some firms try to increase or decrease their short-term earnings by changing required money for retirement allowance or by changing the actuarial assumptions to estimate pension related liability [2–4]. In that context, new estimation method for retirement related liability could be an interesting observation or checkpoint for the transparency of accounting information in Korea. Generally speaking, the adoption of IFRS is supposed to improve the principal qualitative characteristics of accounting information in relevance, reliability, understandability, and comparability [5, 6].

To review the changes in firm's liability for retirement allowance, we select listed companies in Korean stock market, which adopted K-IFRS early in 2009 or 2010. We compare their liability for retirement allowance in financial reports after activation of new policy. Also we check related public announcements from the companies if they had provided proper explanations for their actuarial assumptions to estimate the liability. With the comparisons, we try to identify suspicious companies where the changes in financial report are hardly explained with the public announcements.

The paper proceeds as follows: we review theories on pension accounting and the previous researches on K-IFRS's adoption. This leads to the different expectations for the effects of new pension policy with K-IFRS. Following that, we review reported estimation results on retirement related liability and data analysis.

## 2. Theoretical Background

In 2005, Korean government introduced occupational pension schemes as a retirement allowance by making a law called “Employee Retirement Benefit Security Act.” After having grace periods for the new policy, Korean government requires that all listed companies actually implement it from 2012. At the same time, new financial standards called K-IFRS are activated.

### 2.1. K-IFRS Adoption with Pension Accounting

K-IFRS provides two different basic pension schemes based on how to decide on the total benefit of retirement and who will operate the reserved fund. Defined-contribution (DC) plan is a type of retirement plan in which the employer, employee, or both make contributions to the account for worker's retirement allowance on a regular basis. Only employer's contributions to the account are guaranteed, not the employee's future benefits. Defined-benefit (DB) pension is a type of pension plan in which an employer promises a specified monthly benefit on retirement predetermined by a formula based on the employee's earning history, tenure of service, and age rather than depending directly on individual investment returns. A DB plan is “defined” in the sense that the benefit formula is defined and known in advance, while a DC plan is defined in the sense that the formula for computing the employer's and employee's contributions is defined and known in advance.

For accounting information, DC plan is simple. Firms provide calculated contribution to individuals and report them as retirement allowances. The money in individual accounts should be operated by retirees and the decision on investment risk and investment rewards is placed on each individual. Also all obligations of additional financial report for the operations of the committed fund are on external financial organization. Therefore, firm or employer does not have any other responsibilities for retirement allowance and operational report for the committed fund.

In contrast, DB plan is complicated to get accounting information for the future liability. Firms should commit legally required money to external financial organization based on their estimation for the total pension related liability. Government announced that the required ratio of external severances will be increased gradually, but it is 60% of the reported liability as before the policy changes. Each firm can reserve more money to get additional tax benefit. However, the decision for the additional money amount to reserve is up to the individual firm and related information is guided to be properly announced.

### 2.2. Issues for Future Liability Estimation

Traditionally, DB plan has been popular for retirees because they can expect certain amount of allowance after their retirement. However, during last few decades, DC plan has gained momentum and popularity. By changing DB plan to DC plan, individual firms can typically save a significant amount of money because the benefits afforded by DC plans are typically lower than what is offered by DB plans. However, it may be also criticized as the primary responsibility for preparing for retirement has been removed from employers and placed on employees.

Accounting information of DB plan starts from the estimation of the employee's pension benefit. IFRS suggests projected benefit obligation (PBO) as a standard estimation method to estimate more realistic future liability, which considers all pension benefits for both vested and nonvested employees based on their future compensation [7]. From the estimated future liability, PBO gets the present value of it with a conservative discount factor. Despite its conservative approach to real pension liability, PBO is being criticized for its inaccuracy [8]. Many actuarial assumptions such as life expectancy or compensation increase rate of work as the source of inaccuracy. Also the complexities associated with estimating DB plan liabilities make it difficult to foresee the required current budget for retirement benefit expenditures [1].

The second issue is related to the accounting flexibility for company's DB plan assets and liabilities. For example, FASB 87 in the U.S. allows the off-balance sheet accounting of pension assets and liability amounts. It means that the estimated liability with PBO and employer's contributions are not recorded as a liability or as an asset on the company's balance sheet. Instead, their netted amounts are reported on the company's balance sheet [9]. It makes firm's financial report cannot deliver proper information on actual financial condition of the firm, which may lead investors to erroneous conclusions. Korea had similar issues until the activation of new financial standards. However, recently adopted K-IFRS is not a perfect solution for the issue either. K-IFRS provides the principles for pension accounting and also permits individual firm's arbitrary operations within the principles. If auditing organization does not provide proper guidelines on actuarial assumptions for PBO or other details of pension accounting, we will have other accounting flexibility issues even with new financial standards [10].

### 2.3. Benefits via Tax Concessions versus Manipulated Accounting Information

Occupational pension schemes for worker's retirement have been encouraged via tax concessions in many countries. Korean government also admits tax benefits for whole amount of external reserves committed to financial organization as retirement allowance, while internal reserves for retirement allowance have a certain limitation for tax benefits. The more money a firm commits to external financial organizations, the more tax benefits it gets.

Researches show that firms with good business results have a tendency to increase the reserves for retirement allowance to get more tax benefits [3, 11]. Some results show that it can be a preceding index for good business results. In contrast, firms expecting a bad business result decrease it to keep earnings as much as possible. Because minor changes in accrual assumption can result in big differences in firm's liability estimation, it is possible that firms can adjust the liability with announcements. Currently proper guidelines for the announcement of change of accrual assumptions are suggested but they are not mandatory.

One example of inappropriate use is recently announced for national pension liability in Korea. Korean Ministry of Strategy and Finance announced that the Korean government needs additional fund for government employees' pension. Government had estimated the liability with fixed life expectancy as the result of 2006 and assumed that current employees with insufficient conditions will not be a subject of pension schemes even in future. With the correction of unrealistic assumptions, government announced that new liability in 2013 was increased by 25 billion dollars compared to previous estimation result.

## 3. Expectations versus Actual Results with Pension Accounting

**Figure 1:**
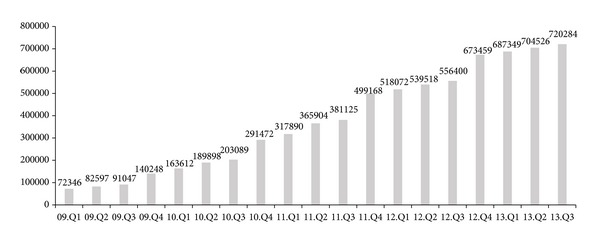
Total amount of external reserves for retirement allowances.

With the legislation of “Employee Retirement Benefit Security Act” in 2005, there were many optimistic forecasts on the size of external reserves for retirement allowance. In 2007, in early stage of the gray period, experts had an expectation that the total reserves will reach up to 80.7 billion dollars for 5 million accounts until 2015. We can see that the actual statistics of the retirement allowance are actually similar to the expectations. Korea has 4.6 million accounts for pension plans, which is 45.6% of whole wage earners in Korea. The external reserves reach 65.5 billion dollars at 2013 Q3.  Figure 1 shows the changes of external reserves for retirement allowances with KRW units.

As we expected, DB plan is the most popular pension plan. 65.1% of the total accounts are for DB plan, while accounts for DC plan are 32.9%, and IRP (Individual Retirement Plans) have 2.0% of the total accounts at 2013 Q3. The firm counts for each pension plan show that 31.6% of total firms provide DB plan only while 52.5% of total firms provide DC plan only. 2.4% of total firms provide both DB and DC plans to their workers and 13.6% of total firms provide IRP.

**Figure 2:**
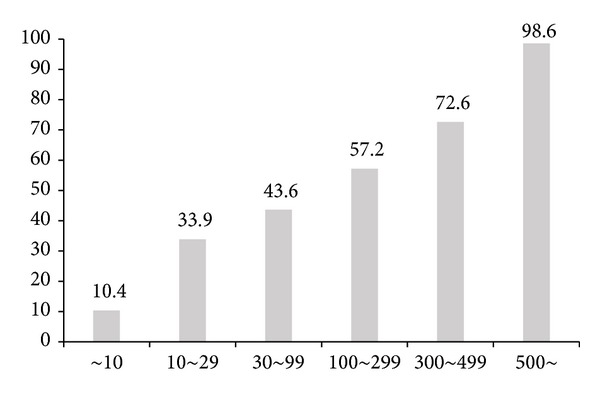
Ratio of firms with pension plan considering its size.

However, the adoption rate and the preference of pension plan can be different considering firm size. As we can see in Figure 2 and Table 1, almost all firms having more than 500 waged workers already adopted pension plan (98.6%) and 49.36% of them provide DB plan only.

### 3.1. Expected Changes in Liability

With the adoption of new financial standards and new retirement policy, there are many different opinions on the change of estimated liability. Simple induction with PBO method leads to the conclusion that it will increase the liability because PBO considers the final wage as bases for liability estimation. Although the estimation is converted to present value with a discount factor, the increase rate of wage is expected to be greater than the inflation rate.

Cho and Rho [12] compared liability based on previous financial standards and K-IFRS with PBO. According to the study, the estimated liability for retirement allowance with PBO will be slightly smaller than the liability with previous financial standard. The firms having actuarial assumptions of higher wage increase rates and lower inflation rates will have estimations with increased liability. It shows that the result of liability estimation depends on individual firm's arbitrary actuarial assumptions and proper regulations are required to prevent manipulating accounting information for pension accounting [2, 8, 13].

For IT industry with higher rate of payroll costs and R&D costs, the firms may prefer DC plan or try to estimate decreased liability.

### 3.2. Financial Report Analysis for Pension Plan

This study chooses the K-IFRS adopted financial reports of 2009 and 2010 for firms listed in Korea stock markets. They provided both financial reports under previous financial reports and K-IFRS, which easily can be compared for the changes in pension related liability caused by PBO. The counts of firms to be compared are 14 from the results of 2009 and 46 from the results of 2010.  Table 2 shows the count of financial reports categorized by its type and year.

We also review their announcement for pension adoption and their actuarial assumptions for their liability estimations. 25 firms from selected 60 had adopted occupational pension plan as retirement benefits and provided financial information K-IFRS requires during the period. DB plan was the most popular plan and 19 firms out of 25 selected it as their pension plan.  Table 3 shows the distribution of firm's pension plans and related liability changes.

For the changes of liability for retirement allowance, 15 firms out of 25 reported that it is decreased compared to other financial reports based on previous financial standards, while 9 firms reported that it is increased. One firm already had external reserves for whole amount of the liability and reported no changes for it.

**Figure 3:**
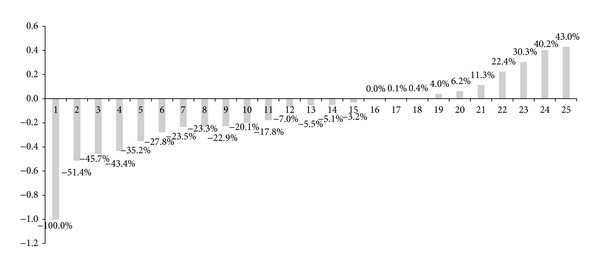
Changed liability for retirement allowance with K-IFRS adoption.

Figure 3 is presenting the change ratio of liability for retirement allowance with K-IFRS. A firm that adopted DC plan and had no external reserves shows 100% decrease of liability. Another firm that already had 100% external reserves for retirement allowances shows no change of liability. Most of their changes are under 20% and match previous expectations.

However, some firms have represented a sharp change above 30% of liability. To track the reasons of individual change, we have to review their actuarial assumptions and previous internal reserves, which should be able to get the information through public announcement.

Reviewing the public announcement related to pension plan and related liability estimation, we just found only 4 disclosures of 3 firms having specific explanation on their assumption. Although K-IFRS recommends public announcements for all pension related information, firms do not have any motivation or obligation for it. Most firms simply described their discount factor to get present value of future pension liability as the market interest rate of government bonds or corporate bonds instead of providing specific number. It shows that current pension accounting guidelines under K-IFRS are not the proper answer to flexible accounting issues [2].

Table 4 summarizes the actuarial assumptions and changes of liability for the cases with public announcements. Each case has significant differences in wage increase rate and discount factor, which are the most influential variables in liability estimation.

Case  1 with relatively higher wage increase rate and lower discount factor resulted in the increase of estimated liability. On the contrary, case 2 with relatively lower wage increase rate and higher discount factor resulted in the decrease of estimated liability. This comparison shows the effect of actuarial assumptions on liability estimation. The issue is that investor cannot judge if the assumptions are appropriate or not.

## 4. Discussion

In this case study, we review the differences of liability for retirement allowance before and after adoption of K-IFRS and pension plan. With the comparison of financial reports for same year company data, we showed that the difference was not recognizable only if the PBO liability estimation is based on reasonable actuarial assumptions. However, some companies used quite different discount factor or wage increase rate and it resulted in big differences in their pension related liability estimation. According to previous research, it can be abused for earning management. Therefore, additional regulations should be added including requirements of proper public announcement for the information.

We hope that future research will continue along the lines of this study by addressing its limitation. This study measured the differences of liability estimation based on individual firm's announcements. Developing more cases with firm internal financial information and developing proper regulations to compare exact financial status of firms especially for liability of retirement allowances could be followed.

## Figures and Tables

**Table 1:** Pension plan considering firm size.

Size	DB	DB/DC	DC	IRP	Firm count
~9	24.97%	1.04%	51.26%	22.73%	140,689
10~29	37.56%	2.54%	59.90%		62,732
30~99	47.15%	4.94%	47.91%		23,542
100~299	54.81%	10.96%	34.22%		6,367
300~499	57.13%	18.59%	24.28%		1,038
500~	49.36%	32.81%	17.83%		1,335

**Table 2:** Financial report counts with early K-IFRS adoption.

Year	Financial report type	Firm count
2009	Consolidated	11
	Individual	3
2010	Consolidated	39
	Individual	7

**Table 3:** Pension plan for firms with early K-IFRS adoption.

Type	Classification	Firm count
Pension plan	DB	19
	DC	3
	DB/DC	3
Changes of liability for retirement allowance	Increase	9
	No change	1
	Decrease	15

**Table 4:** Important actuarial assumptions from public disclosure.

Assumption	Case 1	Case 2	Case 3	Case 4
Discount factor	4.95%	7.37%	6.55%	5.39%
Inflation rate		2.50%	2.50%	
Interest rate	3.50%	5.85%	5.85%	4.48%
Wage increase rate	6.15%	3.79%	3.68%	Standard* + 3.33%
Change of liability	22.38%	−51.36%	N/A	6.21%

*Standard wage increase announced by Korea Insurance Development Institute.
